# Microstructural Changes across Different Clinical Milestones of Disease in Amyotrophic Lateral Sclerosis

**DOI:** 10.1371/journal.pone.0119045

**Published:** 2015-03-20

**Authors:** Francesca Trojsi, Giuseppina Caiazzo, Daniele Corbo, Giovanni Piccirillo, Viviana Cristillo, Cinzia Femiano, Teresa Ferrantino, Mario Cirillo, Maria Rosaria Monsurrò, Fabrizio Esposito, Gioacchino Tedeschi

**Affiliations:** 1 Department of Medical, Surgical, Neurological, Metabolic and Aging Sciences, Second University of Naples, Naples, Italy; 2 MRI Research Center SUN-FISM—Second University of Naples, 80138 Naples, Italy; 3 Department of Neuroscience, University of Parma, 43100 Parma, Italy; 4 Department of Medicine and Surgery, University of Salerno, 84081 Baronissi (Salerno), Italy; University of Ulm, GERMANY

## Abstract

Neurodegenerative process in amyotrophic lateral sclerosis (ALS) has been proven to involve several cortical and subcortical brain regions within and beyond motor areas. However, how ALS pathology spreads progressively during disease evolution is still unknown. In this cross-sectional study we investigated 54 ALS patients, divided into 3 subsets according to the clinical stage, and 18 age and sex-matched healthy controls, by using tract-based spatial statistics (TBSS) diffusion tensor imaging (DTI) and voxel-based morphometry (VBM) analyses. We aimed to identify white (WM) and gray matter (GM) patterns of disease distinctive of each clinical stage, corresponding to specific clinical milestones. ALS cases in stage 2A (i.e., at diagnosis) were characterized by GM and WM impairment of left motor and premotor cortices and brainstem at ponto-mesenchephalic junction. ALS patients in clinical stage 2B (with impairment of two functional regions) exhibited decreased fractional anisotropy (FA) (p<0.001, uncorrected) and increased mean (MD) and radial diffusivity (RD) (p<0.001, uncorrected) in the left cerebellar hemisphere and brainstem precerebellar nuclei, as well as in motor areas, while GM atrophy (p<0.001, uncorrected) was detected only in the left inferior frontal gyrus and right cuneus. Finally, ALS patients in stage 3 (with impairment of three functional regions) exhibited decreased FA and increased MD and RD (p<0.05, corrected) within WM underneath bilateral pre and postcentral gyri, corpus callosum midbody, long associative tracts and midbrain, while no significant clusters of GM atrophy were observed. Our findings reinforce the hypothesis that the neurodegenerative process propagates along the axonal pathways and develops beyond motor areas from early stages, involving progressively several frontotemporal regions and their afferents and efferents, while the detection of GM atrophy in earlier stages and its disappearance in later stages may be the result of reactive gliosis.

## Introduction

Amyotrophic lateral sclerosis (ALS) is the most frequent motor neuron disease characterized by progressive atrophy and weakness of bulbar, limb, and respiratory muscles [1]. Recent studies, including histochemical [2, 3], neuroradiological [4–9] and neuropsychological [10–12] analyses, have demonstrated that a widespread frontotemporal involvement is present in up to 50% of patients, and have suggested that these findings are in line with the broadly described genetic [13, 14] and neuropathological [15, 16] continuum identified between ALS and frontotemporal lobar degeneration (FTLD). Moreover, similarly to what observed in other neurodegenerative diseases characterized by tau and alfa-sinuclein pathology [17–19], there is increasing evidence that in ALS-FTLD spectrum the aggregation of transactive response DNA binding protein 43 KDa (TDP-43) could sequentially disseminate during disease course from a focal site of onset in a prion-like, cell-to-cell manner [20]. This hypothesis recalls the clinico-pathological observation that motor neurons degeneration in ALS starts focally and spreads from motor domains towards several extra-motor regions along anatomical pathways [2, 3, 21, 22]. Furthermore, recent immunohistochemical evidence has depicted the probable sequential pattern of propagation of TDP-43 pathology in ALS, identifying four neuropathological stages of disease progression [23].

In vivo, advanced neuroimaging studies have corroborated the theory of a multisystem degeneration in ALS [4]. In particular, diffusion tensor imaging (DTI) findings of reduced white matter (WM) integrity, in terms of decreased fractional anisotropy (FA) and increased mean (MD) and radial diffusivity (RD) in the frontal, temporal, and parietal lobes and in the corpus callosum (CC) of ALS patients [5,7,8,24], have allowed to suggest a probable linkage between widespread WM involvement and the multi-domain clinical impairment frequently reported in ALS [10–12,25]. Moreover, on the basis of the neuropathological evidence of progressive stages of TDP-43 pathology affecting specific fiber tracts [23], significant differences between ALS patients and controls for these WM fiber bundles have been recently shown in vivo by using a new tract of interest-based fibre tracking approach [26], thereby identifying the DTI correlates of the previously described immunohistochemical stages of disease [23]. With regard to application of brain morphometry to ALS, especially voxel- (VBM) and surface-based (SBM) morphometry, which allowed a fully automated whole-brain measurement of regional brain atrophy [27] and cortical thickness [28], several studies have identified a widespread gray matter (GM) damage in frontal, temporal, parietal and occipital lobes in different clinical stages of disease [6, 8, 9, 29–31]. In addition, some VBM and SBM studies have revealed significant correlations between cognitive impairment and frontotemporal cortical atrophy or thinning in non-demented ALS patients [9, 29–32]. However, the progressive extent and the regional distribution of GM and WM involvement during disease course, a crucial point for assessing ALS pathophysiology, remain still to be elucidated.

The objective of the present cross-sectional study was to investigate DTI and VBM patterns of sequential dissemination of brain pathology across progressive clinical milestones of disease in a large cohort of ALS patients, divided into three subsets according to the staging system for ALS recently proposed by Roche et al. [33]. We applied whole-brain tract-based spatial statistics (TBSS) DTI [34] and VBM analyses to investigate FA, MD and RD changes in combination with GM atrophy assessment across different milestones of disease corresponding to clinical stages, from diagnosis (stage 2A) to evidence of clinical signs of ALS in three functional regions (stage 3). We hypothesized to depict a structural pattern of extent of the neurodegenerative process, consistent with the clinical progression of ALS.

## Methods

### Case selection

Fifty-four right-handed patients (29 M, 25 F; mean age 60.3 ± 11.2), 38 with definite ALS and 16 with probable or probable laboratory-supported ALS, according to diagnostic El-Escorial criteria [35], were consecutively recruited at the First Division of Neurology of the Second University of Naples (Naples, Italy).

Definite, probable or probable laboratory-supported ALS was diagnosed when combined lower and upper motor neuron dysfunction was identified in bulbar and spinal-innervated regions. With regard to clinical phenotypes, according to ALS subtype classification of Chiò et al. [36], forty patients exhibited classic phenotypes, ten bulbar phenotypes, two flail arm phenotypes and two flail leg phenotypes. Patients with major cognitive impairment (e.g., ALS-Dementia, ALS-FTD), progressive bulbar palsy or primary lateral sclerosis were not included.

All patients were classified according to the clinical staging system for ALS proposed by Roche et al. [33], as following: Stage 1: symptom onset (involvement of first region); Stage 2A: diagnosis; Stage 2B: involvement of a second region; Stage 3: involvement of a third region; Stage 4A: need for gastrostomy; Stage 4B: need for respiratory support (non-invasive ventilation). Considering that no patient at onset or with gastrostomy or respiratory support has been enrolled (since the former were less numerous than patients classified in other stages of disease and the latter were unsuitable to be examined by MRI), we defined three subsets of patients in stages 2A, 2B and 3 (i.e., from diagnosis to involvement of three regions), each including eighteen patients (for more details, see Table 1).

**Table 1:** Detailed patients and controls characteristics.

Clinical features	ALS patients (n = 54)	ALS patients with involvement of a first region (stage 2A) (n = 18)	ALS patients with involvement of a second region (stage 2B) (n = 18)	ALS patients with involvement of a third region (stage 3) (n = 18)	Healthy controls (n = 18)
Mean age (years ± SD) (range)	60.3 ± 11.1 (36–80)	59.2 ± 11.6 (36–80)	59.5 ± 10.6 (37–80)	62.4 ± 11.3 (36–74)	61 ± 8.1 (43–76)
Gender (male: female)	29:25	9:9	10:8	11:7	9:9
Mean disease duration (years ± SD) (range)	2.7 ± 1.9 (0.5–7)	2 ± 1.6 (0.5–7)	2.1 ± 1.2 (1–6)	3.9 ± 2.1 (1–7)	-
El Escorial criteria for ALS (definite: probable)	38:16	11:7	13:5	14:4	-
Clinical onset (bulbar: upper limbs: lower limbs)	13:13:28	2:4:12	7:4:7	4:5:9	
ALS FRS-R (mean ± SD) (range)	33.4 ± 7.9 (16–47)	36.3 ± 7.4 (19–47)	32.2 ± 8.6 (16–44)	31.6 ± 7.3 (21–44)	-
UMN score (mean ± SD) (range)	7.2 ± 4.5 (1–16)	7.6 ± 4.3 (2–15)	7.2 ± 4.4 (1–15)	6.7 ± 5 (1–16)	-
ACE-r (cut off 88) (mean ± SD) (range)*	87.5 ± 10.7 (64–99)	90 ± 12.3 (64–99)	87.6 ± 11.8 (65–97)	84.5 ± 6.9 (73–91)	89 ± 2.7 (88–98)
FrSBe scale*					
Total score (mean ± SD)	104.5 ± 24.9	102.1 ± 27.5	105 ± 28.9	107 ± 18	-
Apathy subscore (mean± SD)	37.2 ± 8.4	32.4 ± 8.8	31.4 ± 8.6	32.7 ± 8.4	-
Disinhibition subscore (mean± SD)	31.6 ± 9.8	32.2 ± 9.5	32 ± 12.6	30.4 ± 7.1	-
Executive dysfunctions subscore (mean± SD)	41.2 ± 12.3	38.8 ± 13.9	41.4 ± 13.9	43.8 ± 8.3	-

*available in 39 patients (i.e., 13 patients in stage 2A, 14 in stage 2B and 12 in stage 3)

As clinical parameters, we measured the ALS functional rating scale-revised (ALSFRS-R) score, index of disability status [37], and the upper motor neuron (UMN) score, measure of pyramidal dysfunction through the evaluation of the number of pathologic reflexes elicited from 15 body sites (i.e., glabellum, masseter, and orbicularis oris, biceps, triceps, finger jerks, knee, ankle, and Babinski responses bilaterally) [38]. Respiratory function, measured by forced vital capacity (FVC), was above 70% in all ALS patients and there was no evidence of nocturnal hypoventilation. None of the patients recruited had additional neurological disease or previous mental illness.

All patients included in the study underwent Addenbroke’s Cognitive Examination Revised (ACE-R), a sensitive and specific battery to detect early cognitive impairment and dementia [39]. It contains 5 subscales: attention and orientation, memory, fluency, language and visuospatial, with higher scores denoting preserved cognitive abilities (cut off 88/100). Fifteen patients (four in stage 2A, five in stage 2B and five in stage 3) were not able to perform the test because of speech or manual difficulties. Moreover, behavioral dysfunctions were measured by the Frontal Systems Behavior (FrSBe) Scale [40], a 46-item behavior rating scale for both patients and caregivers, designed to provide a total frontal disturbance score (T score) and three subscale scores (or subscores), which allow to assess apathy, disinhibition, and executive alterations. This scale quantifies behavioral changes over time by combining retrospective and current assessments of frontal dysfunction (T > 65 is defined as impaired behavior and executive functions). In our population we considered T scores and subscores derived from caregivers and referring to the present time and these data were available in thirty-nine patients (Table 1).

Genetic analysis was performed in all patients. Specifically, five patients, one with familial and four with sporadic ALS, were positive respectively for: i) an heterozygous mutation c.149T>C in the exon 5 of the superoxide (SOD1) gene (i.e., a case of familial ALS), ii) abnormal hexanucleotide repeat expansions of C9ORF72 gene (i.e., three sporadic cases affected by classical ALS with behavioral abnormalities), and iii) an heterozygous mutation p.Asn352Ser in the exon 6 of TARDBP gene (i.e., a patient with sporadic ALS characterized by spastic quadriparesis).

Right-handed HCs were enrolled among the non consanguineous caregivers of patients and by word of mouth. They underwent a multidimensional assessment, including neurological examination and a brief neuropsychological evaluation. Eighteen neurologically and cognitively normal subjects (9 M, 9 F; mean age 61 ± 8.1) were included in the study.

### Ethics Statement

The research was conducted according to the principles expressed in the Declaration of Helsinki. Ethics approval was obtained from the Ethics Committee of Second University of Naples. Patient or family written consent was obtained from each participant.

### Imaging acquisition

Magnetic-resonance images were acquired on a 3T scanner equipped with an 8-channel parallel head coil (General Electric Healthcare, Milwaukee, Wisconsin). Whole-brain DTI was performed using a GRE EPI sequence (repetition time = 10000 msec, echo time = 88 msec, field of view = 320 mm, isotropic resolution = 2.5 mm, b value = 1,000 s/mm2, 32 isotropically distributed gradients, frequency encoding RL).

### Diffusion tensor imaging (DTI) analysis

A voxel-based TBSS approach was used for group analysis of DTI data [34]. DTI data sets were processed with the Functional MRI of the Brain (FMRIB) Software Library (FSL) software package (www.fmrib.ox.ac.uk/fsl). Preprocessing included eddy current and motion correction and brain-tissue extraction. After preprocessing, DTI images were averaged and concatenated into 33 (1 B = 0 + 32 B = 1000) volumes and a diffusion tensor model was fitted at each voxel, generating FA, MD, and eigenvalue (λ1, λ2, λ3) maps. The average of the second and third eigenvalues of the diffusion tensor was used for the definition of the RD. Images were warped to the Montreal Neurological Institute (MNI) 152 template, available as standard T1 data set in the FSL software package. TBSS was run with FA maps to create the “skeleton”, which represents the center of all fiber bundles in common to all subjects, and which was used for all other maps. To this purpose, FA images of all subjects (n = 72) were aligned to a common target (1x1x1 mm MNI152 FMRIB58_FA standard space) using nonlinear registration. A mean FA skeleton was then created with threshold of FA>0.2. Moreover, the TBSS results were linked to standard anatomic data derived from the International Consortium of Brain Mapping DTI-81 WM labels atlas (Johns Hopkins University, Baltimore, MD) [41,42].

Individual skeleton images were submitted to a GLM analysis with appropriate design matrices and linear contrasts defined for the group comparisons and the correlations between all diffusivity parameters (FA, RD, MD) and clinical measures of disease duration and disability (ALSFRS-R scores, ACE-R, FrSBe scale T-score). The results of voxel-wise correlations were shown on the skeleton map after correction for multiple comparison with the Threshold_Free Cluster Enhancement (TFCE) technique [34].

### Regional atrophy measurements: voxel-based morphometry (VBM)

We performed a VBM analysis using the VBM8 toolbox (http://dbm.neuro.uni-jena.de/vbm.html) of SPM8 software package (http://www.fil.ion.ucl.ac.uk/spm/) with default parameters incorporating the DARTEL toolbox in order to obtain a high-dimensional normalization protocol [27]. Images were bias-corrected, tissue-classified, and registered by using linear (12- parameter affine) and nonlinear transformations (warping) within a unified model [27]. Subsequently, the warped GM segments were affine-transformed into MNI space and were scaled by the Jacobian determinants of the deformations to account for the local compression and stretching that occurs as a consequence of the warping and affine transformation (modulated GM volumes) [27]. Finally, the modulated volumes were smoothed with a Gaussian kernel of 8-mm full width at half maximum. The GM volume maps were statistically analyzed by using the general linear model based on Gaussian random field theory. Statistical analysis consisted of an analysis of covariance (ANCOVA) with age and sex as covariates of no interest. The MNI152 (a.k.a., International Consortium for Brain Mapping—ICBM—152) atlas [43,44], incorporated into SPM8, was used for localizing anatomically VBM results.

## Results

### Demographics

No significant differences were identified for any of the demographic variables (age and gender) between patient groups and the control cohort (Table 1).

### TBSS DTI analysis

#### Differences between ALS patients and controls

**Fig 1:**
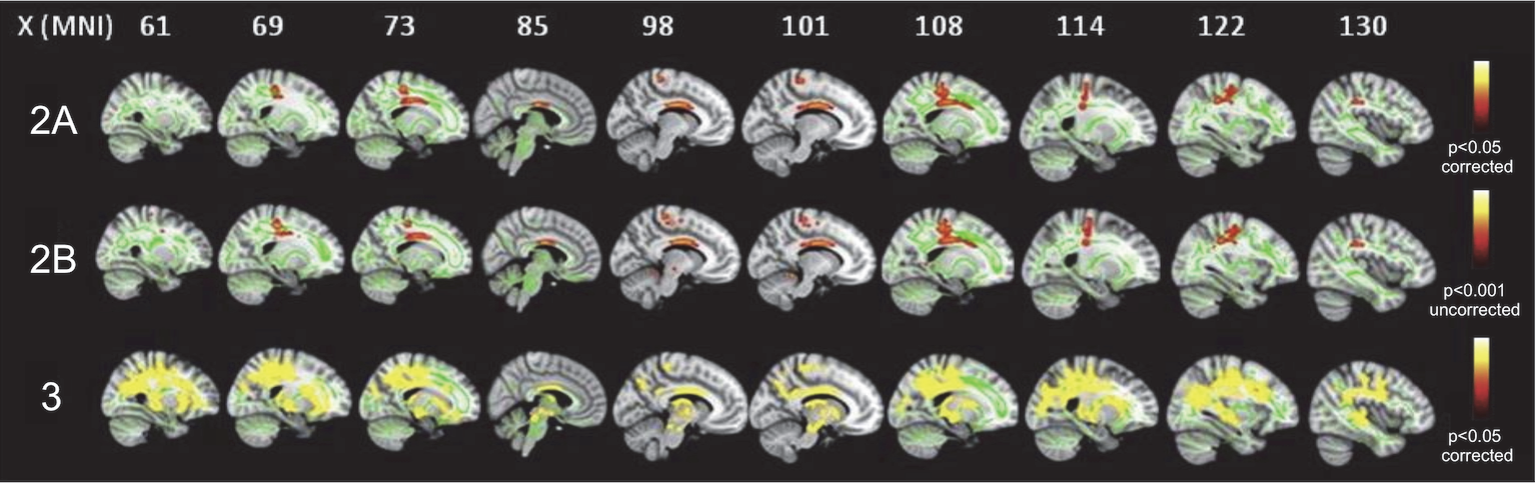
FA differences between ALS patients in the three stages of ALS and healthy controls. in the stage 2A, FA decrease (red-yellow scale, p<0.05, corrected) is evident in the body of CC and left corticospinal tract (CST), principally in its rostral part underneath primary motor cortex (upper panels); in the stage 2B, FA decrease (red-yellow scale, p<0.001, uncorrected) occurs in the left cerebellar hemisphere, beyond CC body and WM underneath the left precentral gyrus (middle panels); in the stage 3, FA decrease (red-yellow scale) (p<0.05, corrected) is highlighted in WM underneath pre and postcentral gyri including the rostral part of CSTs, the body of CC, bilateral superior and inferior longitudinal and inferior fronto-occipital fasciculi (lower panels).

In comparison to HCs, ALS patients in clinical stage 2A (i.e., at diagnosis) exhibited decreased FA (p<0.05, corrected for multiple comparisons) in the body of CC and left corticospinal tract (CST), principally in its rostral part underneath the primary motor cortex (Fig. 1, upper panels; Table 2), while increased MD and RD (p<0.001, uncorrected) was detected in brainstem (at the ponto-mesenchephalic junction), CC and WM underneath left primary motor and premotor cortices.

**Table 2:** Mean values of FA, RD and MD in the volumes of interest (VOIs), derived from Johns Hopkins University (JHU, Baltimore, Maryland) white matter tractography Atlas of FSL [41, 42], which showed significant differences comparing the three groups of patients to healthy controls (HCs).

VOIs (MNI x, y, z)	FA mean values (T values)				RD mean values (T values)				MD mean values (T values)			
	2A	2B*	3	HCs	2A*	2B*	3	HCs	2A*	2B*	3	HCs
CC body 0; −9; 28	0.4889 (2.57951)	0.5077	0.48882 (2.17637)	0.51987	0.00074	0.00072	0.00105 (2.19738)	0.00101	0.0011	0.001	0.00117 (2.03213)	0.00114
Left CST (inferior VOI)** −7; −23; −29	0.54567	0.54627	0.53821 (2.09896)	0.56732	0.00054 (1.4373)	0.00052	0.00055 (2.21023)	0.00051	0.00081 (1.32477)	0.00078	0.00082	0.00079
Left CST (superior VOI) ** −22; −33; 35	0.44143 (2.32483)	0.44909	0.43361 (2.15339)	0.45385	0.00059 (2.7912)	0.00056	0.00060 (2.44686)	0.00056	0.00079 (3.73685)	0.00077	0.00080 (2,31510)	0.00077
Ponto−mesenchephalic junction 2; −28; 36	0.51765	0.50049	0.53165 (1.70243)	0.55187	0.00052 (1.8639)	0.00054	0,00053 (1.789)	0.00048	0.00075 (1.60280)	0.00076	0.00078	0.00074
Left thalamic radiations −8; −9; 3	0.44482	0.45072	0.43890 (1.76817)	0.46144	0.00062	0.00062	0.00065 (1.80531)	0.00061	0.00086	0.00085	0.00087 (1.82558)	0.00084
Right thalamic radiations 7; −9; 4	0.44887	0.46616	0.44470 (1.89831)	0.46963	0.00062	0.00060	0.00065 (1.88179)	0.00061	0.00085	0.00084	0.00088 (1.99424)	0.00085
Left SLF −39; −14; 30	0.37914 (2.48682)	0.38617 (1.68064)	0.37017 (2.04754)	0.39655	0.00062	0.00061	0.00064 (2.1832)	0.0006	0.00078 (3.19022)	0.00077	0.00077 (2.06701)	0.00077
Right SLF 50; −5; 27	0.37978	0.38754	0.37158 (1.99208)	0.39201	0.00061	0.00061	0.00064 (1.98237)	0.00060	0.00078	0.00077	0.00080 (1.92972)	0.00078
Left ILF −30; −69; −3	0.44682	0.44341	0.43250 (1.91777)	0.45772	0.00075	0.00073	0.00079 (1.81535)	0.00074	0.00101	0.00098	0.00104 (1.94866)	0.00100
Right ILF 31; −69; −3	0.46276	0.46223	0.44887 (1.89097)	0.47409	0.00068	0.00066	0.00071 (1.83310)	0.00065	0.00094	0.00091	0.00096 (1.93383)	0.00091
Left IFOF −28; −72; −2	0.38364	0.38461 (1.92436)	0.37350 (1.91386)	0.39329	0.00071	0.00071	0.00074 (1.81398)	0.00070	0.00091	00009	0.00094 (1.88059)	0.00091
Right IFOF 29; 38; 3	0.38069	0.38653	0.39339 (1.79248)	0.39339	0.00070	0.00069	0.00073 (1.7927)	0.00069	0.0009	0.00088	0.00092 (1.87716)	0.00089
Right uncinate fasciculus 27; 13; −7	0.31557	0.31871	0.32374 (1.67958)	0.32374	0.00074	0.00072	0.00077 (1.63183)	0.00074	0.00089	0.00088	0.00093 (1.74693)	0.00090
Amigdala (left) −23; −8; 10	0.17423	0.18179 (2.54173)	0.17768 (1.80887)	0.17582	0.00119 (2.817)	0.00119 (2.8035)	0.00123 (1.59172)	0.00113	0.00130 (1.7234)	0.00130 (2.8803)	0.00135	0.00123
Amigdala (right) 26; −7; −9	0.16707	0.17126	0.16806 (2.25370)	0.17027	0.00133 (3.214)	0.00128 (2.7882)	0.00137	0.00129	0.00144 (2.0019)	0.00139 (2.754)	0.00148	0.00140

T values, when significant, are indicated in brackets.

CC = corpus callosum; CST = corticospinal tract; IFOF = inferior fronto-occipital fasciculus; ILF = inferior longitudinal fasciculus; SLF = superior longitudinal fasciculus; VOI = volume of interest

*T values which resulted significant at an uncorrected level (p<0.001);

**Inferior VOI of CST extend from the pre- and post- central gyri to the cerebral peduncles; superior VOI of CST extend from the cerebral peduncles to the caudal portion of the pons.

Compared to HCs, ALS patients in clinical stage 2B (i.e., with impairment of two functional regions) exhibited decreased FA (p<0.001, uncorrected) in the left cerebellar hemisphere, brainstem precerebellar nuclei and premotor cortex, beyond CC body and WM underneath the left precentral gyrus (Fig. 1, middle panels). Moreover, MD and RD measures were increased (p<0.001, uncorrected) in WM underneath the left precentral gyrus, left and right amygdala, left cerebellar hemisphere and brainstem precerebellar nuclei.

Comparing HCs to ALS patients in clinical stage 3 (i.e., with involvement of three functional regions), we found decreased FA and increased MD and RD (p<0.05, corrected for multiple comparisons) in WM underneath pre and postcentral gyri including the rostral part of CSTs, the body of CC, thalamic radiations, bilateral superior and inferior longitudinal and fronto-occipital fasciculi, right uncinate fasciculus and midbrain (Fig. 1, lower panels; Table 2).

#### Differences between patient groups

Comparing ALS patients in clinical stage 2B to patients in stage 2A, we observed a decreased FA (p<0.001, uncorrected) in the right insula, an increased MD (p<0.001, uncorrected) in the left precentral gyrus and premotor cortex, and an increased MD and RD in the brainstem at the ponto-mesenchephalic junction (p<0.001, uncorrected). Moreover, comparing ALS patients in clinical stage 3 to patients in stage 2B, we found a decreased FA (p<0.001 uncorrected) in the brainstem at the ponto-mesenchephalic junction, superior longitudinal fasciculi, WM underneath precentral and cingulate gyri, and increased MD and RD (p<0.05, corrected for multiple comparisons) bilaterally in thalamic radiations, superior and inferior longitudinal fasciculi, WM underneath precentral gyri, CC body and right uncinate fasciculus.

#### Voxel-wise correlation analysis

**Fig 2:**
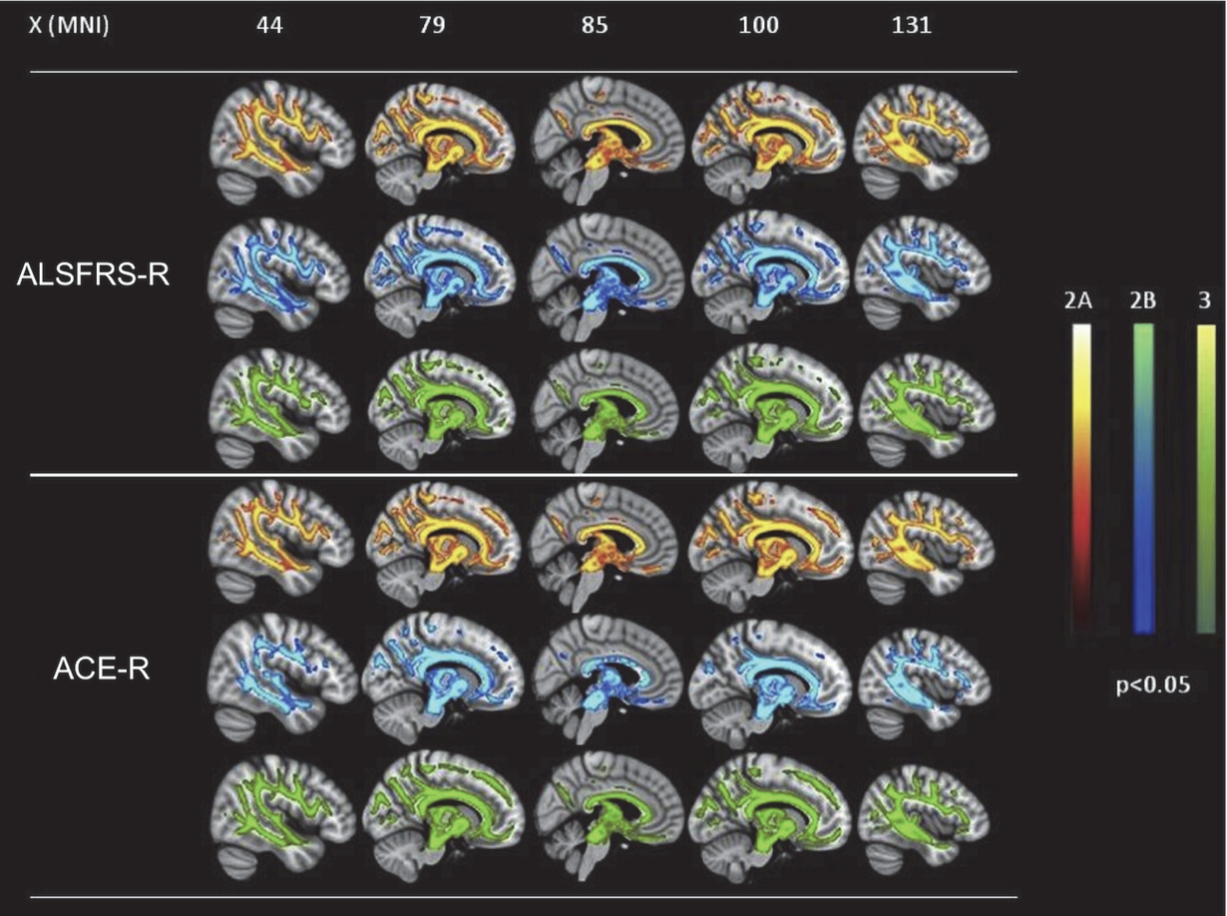
Voxel-wise correlation analyses between ALSFRS-R and ACE-R scores and FA in the three groups of patients. Widespread patterns of positive correlations (p<0.05, corrected) between both disability scores and FA in all clinical stages examined, with overlaps between the three patients groups.

In each clinical stage examined, we detected significant positive correlations between FA and ALSFRS-R and ACE-R scores (p<0.05, corrected for multiple comparisons) in the midpart of CC, superior and inferior longitudinal and fronto-occipital fasciculi and within WM underneath primary motor and premotor cortices, inferior frontal and temporal gyri, supramarginal gyri, visual cortices and brainstem at the ponto-mesenchephalic junction (Fig. 2). Moreover, these widespread patterns of correlation were observed in all clinical stages considered, with an overlapping pattern between the three patients groups. Conversely, no significant correlations were observed between DTI metrics and disease duration in the three subsets of patients evaluated. RD and MD showed positive correlations with FrSBe scale T-scores in all the clinical stages considered in the midpart of CC, superior and inferior longitudinal and fronto-occipital fasciculi and within WM underneath primary motor and premotor cortices, inferior frontal and temporal gyri and brainstem at the ponto-mesenchephalic junction (S1 Fig.).

### VBM analysis

#### Differences with controls

**Fig 3:**
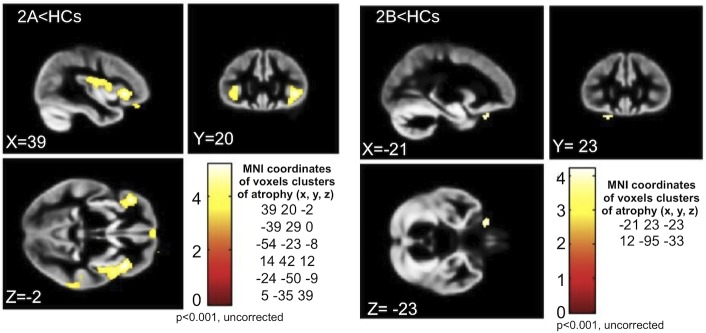
VBM results in 2A and 2B subsets compared to healthy controls. ALS patients in stage 2A show GM atrophy (p<0.001, uncorrected) in the right and left precentral gyri, left inferior frontal gyrus, right anterior cingulate, right medial frontal gyrus, left superior temporal gyrus, left parahippocampal gyrus and right insula (left panels). ALS patients in stage 2B exhibit GM atrophy (p<0.001, uncorrected) only in the left inferior frontal gyrus and right cuneus (right panels).

Comparing all ALS patients to HCs, we observed several voxel clusters of GM atrophy, mainly in the frontal and temporal lobes, although at an uncorrected level of significance (p<0.001): the most atrophic areas were right and left precentral gyri, right medial frontal gyrus, left inferior frontal gyrus, right orbital gyrus, right uvula and right and left insula. Moreover, comparing ALS patients in stage 2A to HCs, we detected GM atrophy (p<0.001, uncorrected) in the right and left precentral gyri, left inferior frontal gyrus, right anterior cingulate, right medial frontal gyrus, left superior temporal gyrus, left parahippocampal gyrus and right insula, while, in comparison to HCs, ALS patients in stage 2B exhibited GM atrophy (p<0.001, uncorrected) only in the left inferior frontal gyrus and right cuneus (Fig. 3). Conversely, comparing ALS patients in stage 3 to HCs, no significant differences in GM atrophy were observed.

#### Differences between patient groups

In comparison to ALS patients in clinical stage 2A, the patients in stage 2B exhibited greater GM atrophy (p<0.001, uncorrected) in the left middle temporal gyrus, right parahippocampal gyrus and left posterior cingulate cortex. Instead, comparing ALS patients in stage 3 to ALS patients in stage 2B, no significant difference in GM atrophy was observed.

## Discussion

Even if ALS is no longer considered as merely a motor disease, the actual spread of the neurodegenerative process throughout the central nervous system has not been fully understood. This cross-sectional study presents the first in vivo evidence of a sequential propagation of structural damage during the clinical course of ALS, from diagnosis to more advanced and progressive milestones of disease. We found a sequential dissemination of WM pathology from motor toward extra-motor areas across different clinical milestones of ALS, with a widespread pathway that may reflect the underlying mechanisms of spreading of the neurodegenerative process. Conversely, GM damage, as detected by using VBM, was principally revealed in both frontal and temporal lobes in less advanced stages of disease, being unapparent in later stages. Probably, this may be interpreted as the radiologic correlate of an early neuronal cells shrinkage and loss, followed by a subsequent reactive gliosis.

We designed our study starting from the neuropathological assumption that ALS-related alterations probably develop consecutively at different central nervous system sites and increase in severity with disease progression. This approach is in line with previous histochemical evidence regarding both ALS and other neurodegenerative disorders [17–19, 45] and with clinical observation of a progressive involvement of different functional sites during ALS evolution, corroborating the hypothesis that ALS-related pathology starts focally and progressively spreads through the anatomy of the motor system towards extra-motor areas [21, 45–47]. Therefore, to investigate in vivo brain structural damage across different stages of disease, we classified the ALS patients enrolled according to a recent staging system for ALS proposed by Roche et al. [33], which allows to identify the progressive clinical involvement of different body sites during disease evolution on the basis of the sequential appearance of signs and symptoms of motor dysfunction identified as progressive milestones of disease.

In our population ALS patients in clinical stage 2A, corresponding to the phase of diagnosis, exhibited FA decrease and/or MD and RD increase in cerebral areas belonging to the motor system, such as WM underneath left primary motor cortex, brainstem at the ponto-mesenchephalic junction, including CSTs, motor nuclei of cranial nerves V and VII, and the midbody of CC, substantially formed by interhemispheric fibers connecting the motor cortices [48]. Moreover, in this stage VBM data showed a widespread frontotemporal GM atrophy, including both motor and extra-motor areas, although at an uncorrected level of significance. These patterns of structural impairment resemble what has been previously described by histochemical analyses in early stages of disease, characterized by extensive accumulation of pathologic TDP-43 aggregates principally in cerebral and spinal motor areas [3, 22, 23]. In fact, early degeneration in ALS has been proven in large Betz and smaller pyramidal neurons which projected their axons to form CSTs [49, 50], with an active and at least partially independent bilateral cortical process of degeneration followed by secondary damage of the CC according to a corticofugal model [17, 46]. Moreover, degeneration of brainstem somatomotor nuclei has been previously described in early stages of ALS [3, 23], also showing significant correlations with clinical appearance of facial and mandibular weakness. In this regard, the small number of subjects with bulbar onset enrolled did not allow us to compare the DTI patterns depicted in these patients to those observed in ALS patients with limbs onset. However, some neuropathological [23] and DTI [51, 52] analyses have reported different patterns of GM and WM damage in cortical and brainstem motor areas when comparing ALS patients with different disease onset.

It is worth noting that in the earliest clinical stage of ALS we found widespread GM and WM alterations, involving several extra-motor frontotemporal regions, such as premotor, prefrontal, anterior cingulate, orbitofrontal and parahippocampal cortices and insular gyri. These results clearly resemble the cognitive and neuropsychiatric changes typical of frontotemporal degeneration and described in ALS patients from early stages of disease [9–12]. Moreover, a number of recent morphometric [9, 30–32, 53] and DTI [54–56] studies, performed in several cohorts of mildly disabled, non-demented ALS patients, have revealed significant correlations between measures of cognitive or behavioral dysfunctions and widespread GM and WM frontotemporal abnormalities, corroborating the evidence that ALS belongs to the disease spectrum of FTLD.

When clinical impairment involved two functional regions, that is in ALS patients in clinical stage 2B, we found that WM damage, corresponding to FA decrease and/or MD and RD increase, showed wider extension to some subcortical areas than in the previous clinical stage. In fact, in the second group of ALS patients compared to HCs we detected some clusters of voxels with decreased FA and increased MD and RD in the WM within left cerebellar hemisphere and brainstem precerebellar nuclei, especially the pontine inferior olivary complex, beyond observing microstructural impairment of CC body and WM underneath left motor and premotor cortices, also described in the previous subset of patients.

With regard to brainstem and cerebellar damage in an intermediate stage of disease, it is to take into account that degeneration of pontine precerebellar nuclei, which receive direct or indirect efferents from the layer V of the agranular neocortex within cortico-pontine-cerebellar or cortico-rubro-olivo-cerebellar tracts, has also been reported in autopsied ALS cases with oculomotor dysfunction, but without axial or limbs ataxia [23, 57, 58]. None of our patients in stage 2B exhibited oculomotor, axial or limbs alterations correlated to cerebellar damage, probably because axial or limbs symptoms may have been masked by predominant spastic and/or atrophic alterations correlated to motor neurons degeneration, while oculomotility pathways have been proven frequently spared in ALS [59].

Notably, increasing neuropathological [60, 61] and MRI [62, 63] evidence lay for a multisystemic neurodegeneration in ALS including damage of several subcortical areas, including cerebellum. Furthermore, some recent studies have revealed that ALS cases associated with repeat expansions in C9ORF72, characterized by phenotypes of frontotemporal dementia with motor neuron disease and without cerebellar signs, have typical cerebellar p62-positive, TDP-43 negative cellular inclusions [60, 61] and show microstructural changes and cortical thinning in the cerebellum [64]. Furthermore, recent functional MRI [62, 65] and 18-fluorodeoxyglucose positron emission tomography [66] findings, derived from investigation of some heterogeneous cohorts of ALS patients, have proven a marked networks hyper-connection and glucose hyper-metabolism spanning subcortical motor areas, principally involving putamen, midbrain, and cerebellum, probably related to an attempt to compensate for the limited primary motor cortex activation. Hence, these results all together confirm that ALS pathology has impact on widespread non-cortical areas whose involvement is not apparent on clinical examination, reflecting the activation of functional plasticity mechanisms in response to the progressive spread of the degenerative process [62].

ALS cases in clinical stage 3, that is patients with involvement of a third functional region, showed the most widespread pattern of WM impairment affecting both motor and extra-motor fiber tracts, such as bilateral CSTs, CC body, bilateral superior and inferior longitudinal and fronto-occipital fasciculi and right uncinate fasciculus. Moreover, comparing ALS patients in this advanced stage of disease to subjects in the previous stage 2B, it is noteworthy that WM of both hemispheres were impaired, with a more marked impairment of long associative tracts. This pattern of spreading of ALS-related pathology towards extra-motor neocortices, such as prefrontal and postcentral cortices, is consistent with the striking clinical evidence of cognitive and behavioral dysfunctions and nociceptive alterations mostly described in ALS patients with severe clinical pictures and longer disease course [12, 67, 68]. Moreover, histochemical data by Brettschneider et al. [23] about dissemination of TDP-43 lesions across brain areas with ongoing disease have revealed a wider spreading of TDP-43 pathology towards prefrontal cortices in advanced ALS patients also affected by executive dysfunctions. In line with these correlations, in patients classified in stage 3 undergone a neuropsychological examination, we observed a trend of decrease of ACE-R scores and increase of FrSBe scale T-scores than in the other subsets of patients, which is indicative of more impaired cognitive and behavioral performances (Table 1).

It is worth noting that, although we showed some correspondence between the progressive extent of microstructural abnormalities across three clinical stages of ALS and the spreading pattern of TDP-43 pathology previously described by Brettschneider et al. [23], we did not aim to explore the DTI and VBM correlates of TDP-43 stages of disease, as performed in a recent fiber tracking analysis by Kassubek et al. [26]. Brettschneider et al. [23] showed the first evidence of the progression of TDP-43 pathology across 4 sequential stages in a large cohort of ALS autopsy cases. Conversely, in our cross-sectional study we analyzed DTI and VBM abnormalities in three groups of phenotypically well-characterized ALS patients who exhibited different milestones of ALS progression, observing a sequential pattern of microstructural damage which seems to resemble the process of disease spreading previously hypothesized at an anatomical level [21].

With regard to VBM results, widespread frontotemporal GM atrophy was observed in all ALS patients compared to HCs, but, comparing each subset of patients to HCs, atrophic GM areas appeared to be progressively less numerous and widespread from stage 2A to stage 3. Specifically, comparing patients in clinical stage 3 to HCs, we found no significant differences in GM atrophy. A similar decreasing ability of measuring GM atrophy from less to more advanced stages of ALS has been reported in previous neuropathological [69, 70] and morphometric [29, 71, 72] studies, which emphasized the limitations of assessing longitudinal measures of cortical atrophy or thinning in ALS, especially within cortical motor areas. However, a recent 6-monthly longitudinal MRI analysis by Menke et al. [31], performed in a large cohort of sporadic ALS patients, has revealed a limited progression of WM pathology in contrast with increasing GM changes particularly in subcortical areas, including basal ganglia. A possible explanation of these inconsistent results about longitudinal GM measures may derive from the occurrence of an hypothetical “ceiling phenomenon” in more advanced stages of disease related to reactive gliosis in the deep layers of the motor cortex, which could “mask” tissue loss, especially in the motor cortex [69, 70]. Moreover, Kwan et al. [73], who examined longitudinal changes of GM volume and cortical thickness in a cohort of 9 ALS and 12 primary lateral sclerosis (PLS) patients, showed that disease duration may influence these longitudinal measures, revealing a greater rate of cortical thinning in ALS patients with shorter disease course. Therefore, it may be hypothesized that in ALS cortical damage occurs in a non-linear fashion, showing an early rapid phase of decline, detected in patients with faster disease evolution, followed by a longer, slower phase of cortical thinning, which may characterize patients with longer disease course. This theory could be helpful to explain our observation of no significant GM atrophy in the most advanced stage of disease, considering that ALS patients with longer disease duration were more represented in the subset of patients in stage 3 (Table 1).

With regard to correlation analysis between clinical features and DTI parameters, we identified significant correlations between FA and both global and cognitive disability scores (i.e., ALSFRS-R and ACE-R) in motor (i.e., WM underneath primary motor cortices, CSTs at the ponto-mesenchephalic junction, midpart of CC) and extra-motor regions (i.e., WM underneath premotor and visual cortices, inferior frontal and temporal gyri, supramarginal gyri, and superior, inferior longitudinal and fronto-occipital fasciculi), as well as between MD and RD and FrSBe scale T-scores. Remarkably, in all clinical stages examined we observed similar widespread patterns of voxelwise correlations. These data are consistent with previous correlation analyses between diffusivity parameters and clinical measure of disease severity and progression performed in heterogeneous cohorts of ALS patients [7, 63, 74]. However, our results suggest that ALSFRS-R, ACE-R and FrSBe total scores, though determinant to characterize disability degree and cognitive status, do not allow to identify different stages of structural brain damage during the disease course. To note, cognitive abnormalities have not been considered for establish an ALS staging system and clinical scores alone, such as disease duration and progression rate or ALSFRS-R score, have been shown lacking to define possible milestones for ALS staging. Specifically, disease progression over time has been proven to be not linear, but curvilinear [75], and ALSFRS- R scale, which measures a single aggregate score related to many different modalities indicative of functional progression, does not include milestones [37]. Conversely, the staging system for ALS proposed by Roche et al. [33], based on simple clinical milestones of the natural history of ALS, was particularly useful for classifying the patients examined in clinical stages, each one of which reflecting the increasing severity of the disease. Interestingly, the King's system proposed by Roche et al. [33] is easy to use because it corresponds both to information discovered by the neurologist and symptoms reported by the patient, simply referring to the detection of neurological weakness in one or more of three commonly body sites explored (i.e., bulbar, cervical and lumbar). However, a possible limitation of such classification may be related to the different clinical consequences of impairment of different sites (especially with regard to bulbar or diaphragmatic involvement), although clinical progression, linked to degeneration of respiratory or swallowing function, implies the classification in the highest stage of disease.

This study has a major limitation related to sample size and characteristics of patients studied. In fact, the recruitment of ALS patients in three consecutive, intermediate stages of disease, although useful for depicting DTI and VBM structural patterns of abnormalities across most part of the ALS course, unavoidably hindered to investigate the whole spectrum of disease which would have required the inclusion of patients at onset or later stages of disease. Moreover, the sample of patients investigated was relatively small, although comparable with those analyzed in previous DTI studies [5,7,8,24, 56,76]. As a consequence, DTI and VBM results might not survive the correction for multiple comparisons, leading to the risk of false-positive results. Furthermore, we cannot exclude that neuroimaging findings may result borderline because of confounding factors, such as clinical and pathological heterogeneity, typically underlying the ALS-related disease process [1]. Another relevant major limitation of our study is linked to the cross-sectional design of our analysis, which allowed only to compare at one time different clinical stages of disease from different groups of patients, instead of performing longitudinal exams. However, it is to take into account that MRI longitudinal studies have been proven quite difficult to be performed for longer periods than 12 months [31, 63, 76], probably because of the severe clinical progression typical of the disease, which makes arduous to execute repeated MRI exams.

In conclusion, our findings imply that microstructural alterations disseminate in a sequential manner across different clinical stages of ALS, helping to expand our understanding of the progressive spread of neurodegeneration. However, the patterns observed could not reflect exactly the pathological process underlying disease evolution, also considering the limitations derived from a relatively small number of subjects examined and the cross-sectional design of the study. In fact, although our results indicate a gradual dissemination of ALS-related pathology from motor towards extra-motor areas across different clinical stages of disease, the biochemical mechanisms involved in dissemination of the neurodegenerative process are still to be clarified.

## Supporting Information

S1 Fig Voxel-wise correlation analysis between FrSBe scale T-scores and RD and MD measures. Widespread positive correlations (p<0.05, corrected) between FrSBe scale T-scores and RD and MD in all clinical stages examined, with overlapping patterns between the three patients groups.
(JPG)
