# Supplementary figures and images for: Microstructural Changes across Different Clinical Milestones of Disease in Amyotrophic Lateral Sclerosis
Source: PLoS One. 2015 Mar 20;10(3):e0119045. doi: 10.1371/journal.pone.0119045 (PMC4368555; doi:10.1371/journal.pone.0119045)

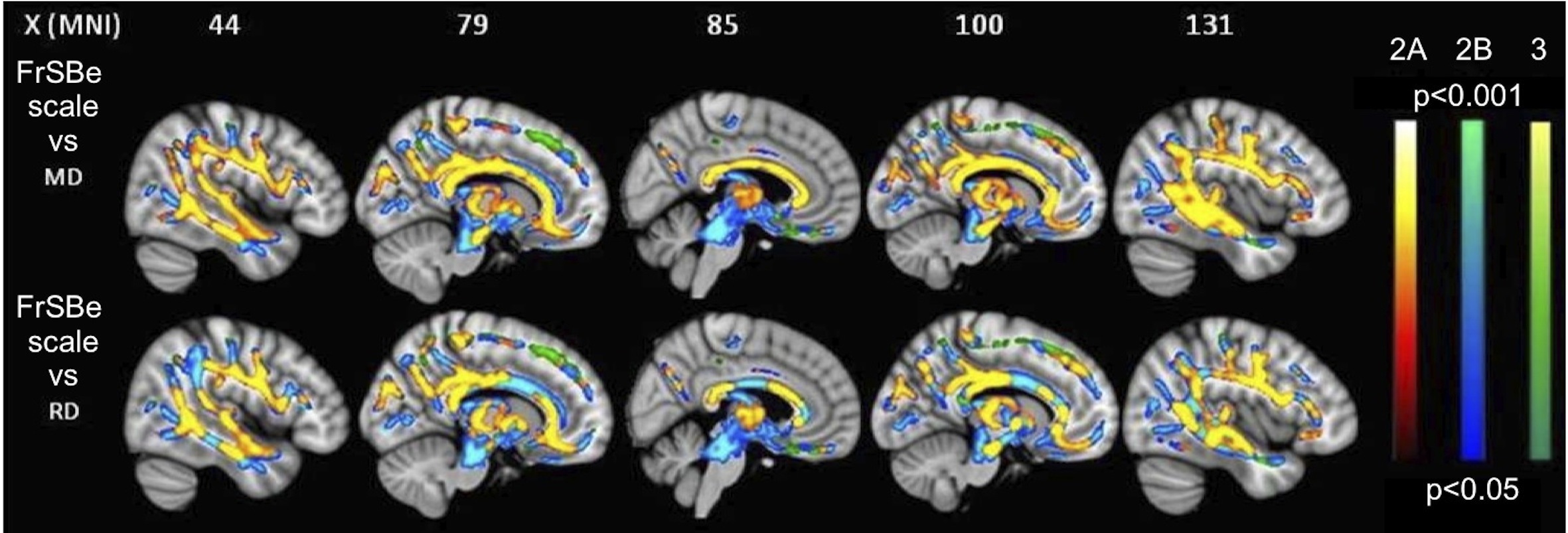

Supplement: S1 Fig — Widespread positive correlations (p<0.05, corrected) between FrSBe scale T-scores and RD and MD in all clinical stages examined, with overlapping patterns between the three patients groups. (JPG) [file pone.0119045.s001.jpg]
